# Supplementary material for: Generalized and scalable trajectory inference in single-cell omics data with VIA
Source: Nat Commun. 2021 Sep 20;12:5528. doi: 10.1038/s41467-021-25773-3 (PMC8452770; doi:10.1038/s41467-021-25773-3)
Supplement: Supplementary file 3 — Reporting Summary [file 41467_2021_25773_MOESM3_ESM.pdf]

nature research

Corresponding author(s): Kevin K. Tsia
Last updated by author(s): Aug 18, 2021

Reporting Summary

Nature Research wishes to improve the reproducibility of the work that we publish. This form provides structure for consistency and transparency in reporting. For further information on Nature Research policies, see our Editorial Policies and the Editorial Policy Checklist.

Statistics

For all statistical analyses, confirm that the following items are present in the figure legend, table legend, main text, or Methods section.

n/a	Confirmed	
☐	☒	The exact sample size (n) for each experimental group/condition, given as a discrete number and unit of measurement
☒	☐	A statement on whether measurements were taken from distinct samples or whether the same sample was measured repeatedly
☒	☐	The statistical test(s) used AND whether they are one- or two-sided<br>*Only common tests should be described solely by name; describe more complex techniques in the Methods section.*
☒	☐	A description of all covariates tested
☐	☒	A description of any assumptions or corrections, such as tests of normality and adjustment for multiple comparisons
☐	☒	A full description of the statistical parameters including central tendency (e.g. means) or other basic estimates (e.g. regression coefficient) AND variation (e.g. standard deviation) or associated estimates of uncertainty (e.g. confidence intervals)
☒	☐	For null hypothesis testing, the test statistic (e.g. F, t, r) with confidence intervals, effect sizes, degrees of freedom and P value noted<br>*Give P values as exact values whenever suitable.*
☒	☐	For Bayesian analysis, information on the choice of priors and Markov chain Monte Carlo settings
☐	☒	For hierarchical and complex designs, identification of the appropriate level for tests and full reporting of outcomes
☒	☐	Estimates of effect sizes (e.g. Cohen's d, Pearson's r), indicating how they were calculated

Our web collection on statistics for biologists contains articles on many of the points above.

Software and code

Policy information about availability of computer code

Data collection	Matlab 2018a
Data analysis	Python 3.7.4 Python modules: palantir 0.2.5, stream 1.0, scvelo 0.2.2, scanpy 1.6.0, cellrank 1.3.1, Slingshot 1.8.0, Seurat 3.2.2, SingleCellExperiment 1.12.0, SingleR 1.4.0, Monocle3 0.2.3.0<br>Custom software code pyVIA 0.1.6

For manuscripts utilizing custom algorithms or software that are central to the research but not yet described in published literature, software must be made available to editors and reviewers. We strongly encourage code deposition in a community repository (e.g. GitHub). See the Nature Research guidelines for submitting code & software for further information.

Data

Policy information about availability of data

All manuscripts must include a data availability statement. This statement should provide the following information, where applicable:

- Accession codes, unique identifiers, or web links for publicly available datasets
- A list of figures that have associated raw data
- A description of any restrictions on data availability

Pancreatic data: Gene Expression Omnibus (GEO) under accession code GSE132188.
Cardiac progenitor data is available from the ENA repository under the accession code PRJEB23303 or from [https://github.com/loosolab/cardiac-progenitors].
B-cell: STATegraData GitHub repository.
Mass cytometry mesoderm: Cytobank [https://community.cytobank.org/cytobank/experiments/71953].
Raw and processed data for scRNA-seq Human Hematopoeisis are available through the Human Cell Atlas data portal at https://data.humancellatlas.org/explore/projects/091cf39b-01bc-42e5-9437-f419a66c8a45.

Embryoid Body: Mendeley Data repository at https://doi.org/10.17632/v6n743h5ng.1.
Mouse Organogenesis : NCBI Gene Expression Omnibus under accession number GSE119945
FACED cell cycle: https://github.com/ShobiStassen/VIA and on FigShare https://doi.org/10.6084/m9.figshare.13601405.v1
scATAC-seq Hematopoiesis: GEO: GSE96772. Processed scATAC-seq data, which include PC values and TF scores per cell can be found in Data S1. of https://doi.org/10.1016/j.cell.2018.03.074
Toy Data: https://github.com/ShobiStassen/VIA

Field-specific reporting

Please select the one below that is the best fit for your research. If you are not sure, read the appropriate sections before making your selection.

☒ Life sciences ☐ Behavioural & social sciences ☐ Ecological, evolutionary & environmental sciences

For a reference copy of the document with all sections, see nature.com/documents/nr-reporting-summary-flat.pdf

Life sciences study design

All studies must disclose on these points even when the disclosure is negative.

Sample size	The number of captured cell images depends on the actual cell count in the culture flask and also the speed of data acquisition of our imaging system under the configuration of fast microfluidic flow. The sample size exceeds 1000 for each experiment, it is large enough to justify statistical analysis
Data exclusions	Images which were out-of-focus, cell-free, or with background noise only were excluded.
Replication	Replications were successfully performed by doing the same cell cycle experiment (including analysis) on two different cell types (MDA-MB231 and MCF7) and the resulting analysis is shown in the manuscript.
Randomization	The cells were randomly extracted from the cell culture samples. The cells are taken our of the culture flasks and put into test tubes (suspended in PBS). During the experiment the cells are pumped into the microfluidic chip for imaging flow cytometry. Because an oscilloscope was used, the image capture is not continuous, and therefore we are effectively sampling the cells the flow through the imaging region.
Blinding	The investigator was blinded to the cell cycle stage of individual cells during the data acquisition and collection procedure. The ground truth was later determined by the fluorescence label.

Reporting for specific materials, systems and methods

We require information from authors about some types of materials, experimental systems and methods used in many studies. Here, indicate whether each material, system or method listed is relevant to your study. If you are not sure if a list item applies to your research, read the appropriate section before selecting a response.

Materials & experimental systems

n/a	Involved in the study	
☒	☐	Antibodies
☐	☒	Eukaryotic cell lines
☒	☐	Palaeontology and archaeology
☒	☐	Animals and other organisms
☒	☐	Human research participants
☒	☐	Clinical data
☒	☐	Dual use research of concern

Methods

n/a	Involved in the study	
☒	☐	ChIP-seq
☒	☐	Flow cytometry
☒	☐	MRI-based neuroimaging

Eukaryotic cell lines

Policy information about cell lines

Cell line source(s)	Breast cancer cell lines: MCF7 and MDA-MB231 (Supplier: ATCC, US).
Authentication	Cellular morphology are routinely checked during cell culture under light microscope prior to imaging experiments.
Mycoplasma contamination	Prevention of mycoplasma contamination was done by adding antibiotic-antimycotic during cell culture.
Commonly misidentified lines (See ICLAC register)	No commonly misidentified lines were used.
